# RhoC and ROCKs regulate cancer cell interactions with endothelial cells

**DOI:** 10.1016/j.molonc.2015.01.004

**Published:** 2015-01-22

**Authors:** Nicolas Reymond, Jae Hong Im, Ritu Garg, Susan Cox, Magali Soyer, Philippe Riou, Audrey Colomba, Ruth J. Muschel, Anne J. Ridley

**Affiliations:** ^1^ King's College London, Randall Division of Cell and Molecular Biophysics, New Hunt's, House, Guy's Campus, London SE1 1UL, UK; ^2^ Gray Institute for Radiation Oncology and Biology, University of Oxford, Oxford OX3 7IJ, England, UK

**Keywords:** Rho GTPases, Endothelial cells, cancer metastasis, RhoC, ROCKs, cell migration

## Abstract

RhoC is a member of the Rho GTPase family that is implicated in cancer progression by stimulating cancer cell invasiveness. Here we report that RhoC regulates the interaction of cancer cells with vascular endothelial cells (ECs), a crucial step in the metastatic process. RhoC depletion by RNAi reduces PC3 prostate cancer cell adhesion to ECs, intercalation between ECs as well as transendothelial migration in vitro. Depletion of the kinases ROCK1 and ROCK2, two known RhoC downstream effectors, similarly decreases cancer interaction with ECs. RhoC also regulates the extension of protrusions made by cancer cells on vascular ECs in vivo. Transient RhoC depletion is sufficient to reduce both early PC3 cell retention in the lungs and experimental metastasis formation in vivo. Our results indicate RhoC plays a central role in cancer cell interaction with vascular ECs, which is a critical event for cancer progression.

## Highlights

- RhoC acts in cancer cells to mediate their interaction with endothelial cells in vitro and in vivo.
- RhoC regulates cancer cell attachment to and insertion between endothelial cells prior to transendothelial migration.
- The RhoC effectors ROCK1 and ROCK2 act similarly to regulate cancer cell interaction with endothelial cells.
- Transient RhoC depletion is sufficient to reduce cancer cell survival in lung blood vessels and metastasis formation.

**Abbreviations**
EC: endothelial cell
HUVECs: human umbilical vein endothelial cells
CFSE: carboxyfluorescein diacetate succinimidyl ester

## 1 Introduction

Metastasis is the formation of secondary tumour foci in organs distant from the original primary tumour. Metastases are usually difficult to treat with current therapies and are responsible for around 90% of human cancer deaths (Gupta and Massague, 2006). To metastasize, cancer cells that have shed from a primary tumour invade their surrounding tissues, then enter the circulation directly through blood or lymphatic vessels (Dadiani et al., 2006; Li et al., 2000; Wyckoff et al., 2000). If they survive in the circulation and attach to blood vessel walls, they can eventually exit the bloodstream through a process called extravasation (Im et al., 2004; Ito et al., 2001; Kienast et al., 2010; Naumov et al., 1999; Reymond et al., 2013; Tsuji et al., 2006; Wang et al., 2004). Depending on the cancer origin and the target organ, tumour cells display different metastatic behaviours. They can extravasate as single cells or initially proliferate in blood vessels and then extravasate (Al‐Mehdi et al., 2000; Gassmann et al., 2009; Martin et al., 2010). Micro‐metastases that survive and proliferate within this new environment then form macroscopic tumours in different organs or tissues (Chambers et al., 2002; Joyce and Pollard, 2009; Nguyen et al., 2009).

Members of the Rho GTPase family control cell adhesion and motility through actin cytoskeleton reorganization, actomyosin contractility and microtubule dynamics. They thereby influence a broad range of processes such as cell movement and cell polarity (Ridley, 2001, 2006, 2008). Activating mutations in Rho GTPases have recently been described in human cancers (Machesky and Sansom, 2012) as well as in some of their effectors such as the kinase ROCK1 (Lochhead et al., 2010). In addition, Rho GTPase expression levels are often significantly different in tumours and metastases compared to surrounding normal tissues and this often correlates with a poor prognosis (Kusama et al., 2006; Rathinam et al., 2011; Vega and Ridley, 2008). RhoA and RhoC, as well as ROCKs, have been directly implicated in the metastasis process *in vitro* and *in vivo*, although the precise steps that they regulate have not been defined (Clark et al., 2000; Croft et al., 2004; Itoh et al., 1999; Ridley, 2013; Somlyo et al., 2000). High expression levels of RhoC correlate with clinical cancer metastasis (Horiuchi et al., 2003; Shikada et al., 2003; Suwa et al., 1998; van Golen et al., 2000). RhoC was one of several Rho GTPases that we found to regulate adhesion of cancer cells to ECs (Reymond et al., 2012a).

Here we investigate the role of RhoC during cancer cell interactions with ECs *in vitro* and *in vivo*. We demonstrate that RhoC is important for cancer cell‐EC 7interactions and could thereby contribute to metastasis.

## 2 Materials and methods

### 2.1 Cell culture and reagents

Primary human umbilical vein endothelial cells (HUVECs) (Lonza) and PC3 cells were grown as previously described (Reymond et al., 2012, 2012).

For western blotting, primary antibodies were used at a dilution of 1:1000 and secondary HRP‐conjugated mouse or rabbit antibodies (Amersham) at 1:5000. The following antibodies were used: RhoC (C‐16, Santa Cruz Biotechnology or D40E4, Cell Signalling), ROCK1 and ROCK2 (mouse, BD Transduction Laboratories), VE‐cadherin (clone 75, BD Biosciences), PECAM‐1 (clone JC70A, Dako), PE‐PECAM‐1 (clone 390, BioLegend), pThr18/Ser19‐MLC2 (#3674, Cell Signalling), MLC2 (#3672, Cell Signalling), and GAPDH (Millipore). HRP‐conjugated antibodies were detected with chemiluminescence reagent (Pierce). TRITC‐conjugated phalloidin (1:400; Invitrogen) were used to detect F‐actin. Where indicated, PC3 cells were labelled with 2 μM 5‐(and‐6)‐carboxyfluorescein diacetate succinimidyl ester (CFSE; Molecular Probes) in RPMI containing 0.1% FCS.

### 2.2 Cell transfection and western blotting

siRNAs were obtained from Dharmacon (Thermo Scientific) or Sigma–Aldrich (sense strands are listed): RhoC‐1 (AUAAGAAGGACCUGAGGCA), RhoC‐2 (GGAUCAGUGCCUUUGGCUA), ROCK1‐1 (GAAGAAACAUUCCCUAUUC), ROCK1‐2 (GAGAUGAGCAAGUCAAUUA), ROCK2‐1 (GCAAAUCUGUUAAUACUCG), ROCK2‐2 (CAAACUUGGUAAAGAAUUG), and non‐targeting control siRNA from Dharmacon (D‐001810‐01) or Sigma–Aldrich (UAGCGACUAAACACAUCAA). PC3 cells (1.25 × 10^5^) were plated in 6‐well dishes and transfected after 24 h with individual siRNA oligos (100 nM) with Optimem‐I and Oligofectamine (Invitrogen). After 72 h, cells were detached from culture plates with non‐enzymatic cell dissociation solution (Sigma–Aldrich) and used for functional assays as described. For western blotting, cells were lysed by scraping into sample buffer (NuPAGE 4× SDS sample buffer; Invitrogen), proteins separated using precast NuPAGE 4–12% Bis‐Tris gels (Invitrogen), transferred to nitrocellulose membrane (Immobilon), and incubated with antibodies in Tris‐buffered saline containing 5% non‐fat milk and 0.1% Tween‐20. For phospho‐MLC analysis, cells were transferred to RPMI containing 1% FCS, 24 h before lysis. Cells were lysed in lysis buffer (80 mM Tris pH 7.5; 10% Glycerol; 2% SDS; 1 mM DTT; 10 mM NaF; 10 mM sodium β‐glycerol phosphate; 1 mM sodium vanadate; 0.5 µM PMSF and protease inhibitor cocktail (Roche)) and immediately snap‐frozen on dry‐ice. Lysates were sonicated for 20 s and centrifuged for 30 min. Supernatants were collected and 4× SDS sample buffer (Invitrogen) was added. Proteins were separated and blotted as above. All primary antibodies were used at 1:1000 and secondary antibodies at 1:2000. Bound antibodies were visualised with horseradish peroxidase‐conjugated goat anti‐IgG antibodies and enhanced chemiluminescence (ECL; Amersham Pharmacia Biotech).

### 2.3 Adhesion assay to ECs

As previously described (Reymond et al., 2012, 2012), CFSE‐labelled PC3 cells (2 × 10^4^) were added for 15 min at 37 °C in RPMI containing 1% FCS to confluent HUVECs grown on a 96‐well dish. Cells were then washed with PBS (Gibco). Adherent cells were quantified with a Fusion α‐FP plate reader (PerkinElmer) using an excitation of 485 nm and an emission filter of 535/25 nm. Fusion 4.02 software and Microsoft Excel were used to acquire raw data and process them, respectively.

### 2.4 Transendothelial migration assay

As previously described (Reymond et al., 2012, 2012), HUVECs were plated onto 10 μg/ml fibronectin‐coated Costar transwells (8‐μm pore size and 6.5‐mm diameter) at 5 × 10^4^ cells/well. Hepatocyte growth factor (40 ng/ml) was added as a chemo‐attractant in the lower chamber before adding 2.5 × 10^4^ CFSE‐labelled PC3 cells. Cells were allowed to transmigrate for 8 h at 37 °C. PC3 cells were recovered from the bottom of the filter and the well, resuspended in PBS containing 5% FCS and counted by flow cytometry (FACSCalibur 3.7, BD Biosciences). Results were processed using Cell Quest software.

### 2.5 Immunofluorescence

HUVECs were grown to confluency on 13‐mm diameter glass coverslips. CFSE‐labelled PC3 cells (2.5 × 10^4^) were added and were fixed at different time points with 3.7% paraformaldehyde in PBS for 20 min. After blocking aldehydes with 50 mM NH_4_Cl for 10 min at room temperature, cells were permeabilized with 0.1% Triton X‐100 for 5 min at 4 °C and then blocked with 5% FCS in PBS for 20 min. Samples were incubated with primary antibodies for 60 min and then with Alexa Fluor 488, 543, and 647 secondary antibodies (Molecular Probes) or dyes. Samples were mounted onto slides with mounting medium (Dako), and images acquired using a Zeiss LSM 510 confocal microscope with a 40× objective and Zen software (Zeiss). Images were processed using Adobe Photoshop software.

### 2.6 Timelapse microscopy and intercalation quantification

As previously described (Reymond et al., 2012, 2012), CFSE‐labelled PC3 cells (3 × 10^4^) were added to confluent HUVECs on 24‐well plates. Cells were monitored by time‐lapse microscopy for up to 5 h in a humidified chamber at 37 °C and 5% CO_2_ with a TE2000 Nikon inverted microscope equipped with a motorized stage (Prior) with a 10× or a 20× objective using Metamorph software (Molecular Devices). To quantify intercalation, a cell was considered as intercalated when its shape was not round, it was no longer phase‐bright and when it was clearly part of the EC monolayer. Cells were tracked manually using ImageJ software to measure their migration speed and the migration distance before intercalation.

### 2.7 Visualization of cancer cell attachment in lung blood vessels

We observed and imaged fluorescently labelled tumour cells and ECs *in situ* in isolated, ventilated blood‐free lungs of SCID mice (6–8‐week‐old female mice) by confocal microscopy as previously described (Im et al., 2004; Reymond et al., 2012a). YFP‐PC3 cells were transfected with a control siRNA and CFP‐PC3 cells were transfected with the RhoC‐1 siRNA, or vice versa. 72h after transfection, both populations were injected in the vena cava (10‐min time‐point) or in the tail vein (6 h and 24 h time‐points) of mice. Blood vessels were stained with a PE‐conjugated mouse anti‐PECAM‐1 antibody injected in the vena cava 5 min before lung dissection. Images of PC3 cells and vascular lung ECs were acquired using a LSM 710 Zeiss confocal microscope using laser excitation at 405 nm (CFP), 488 nm (YFP) and 543 nm (PE) with a 20× (quantification experiments) or a 40× (morphology experiments) objective. The morphological analysis was carried out only on single cells or groups of 2 cells. Images were acquired from at least three independent transfections of PC3 cells with siRNAs. At least 50 cells per condition were analysed from at least 3 mice per condition. It was not possible to carry out statistical analysis because of the variability in the number of cells that could be analysed in each mouse. 2‐dimensional and 3‐dimensional images were processed using Adobe Photoshop and Amira software.

### 2.8 Lung metastasis assay

PC3 cells were transfected with a non‐targeting siRNA (Control) or siRNA RhoC‐1. After 72 h, cells were detached from culture plates by incubation in nonenzymatic cell dissociation solution (Sigma–Aldrich), and 10^6^ cells exhibiting 90% viability (Roche Casy Cell Counter) were suspended in 200 μl of serum‐free RPMI before injection into the tail veins of SCID mice (6–8‐week‐old female mice). After 6 weeks, lungs were analysed for the presence of metastatic foci.

### 2.9 Statistical analysis

Each condition was performed in triplicate and experiments were all performed at least 3 times. Data are expressed as means ± s.e.m. Statistical significance of *in‐vitro* assays were determined by Student's t‐test unless otherwise indicated. Statistical significance of *in vivo* assays was determined by two‐way ANOVA. For PECAM‐1 staining around cancer cells, Kolmogorov–Smirnov tests on pairs of distributions were done. In all analyses, differences were considered statistically significant at p < 0.05.

## 3 Results

### 3.1 RhoC is required for cancer cell intercalation and transendothelial migration between ECs

In an RNAi screen, we recently showed that depletion of several Rho GTPases, including RhoC, reduces adhesion to ECs (Reymond et al., 2012a). RhoC has recently been reported to affect cancer cell TEM (Brown et al., 2014), but the steps at which it regulates cancer cell interaction with ECs is not known. We therefore investigated how RhoC affects different steps of cancer cell: EC interaction.

Interactions between cancer cells and vascular ECs during the process of TEM were investigated by adding prostate cancer cells to confluent human umbilical vein endothelial cells (HUVECs) as previously described (Reymond et al., 2012b). Briefly, PC3 prostate cancer cells adhere to ECs within 15 min then move on top of them to reach EC junctions (Movie S1). They form protrusions that extend between EC junctions and spread between ECs as early as 30 min after attachment by inducing endothelial retraction. We have named this process cancer cell intercalation (Reymond et al., 2012, 2012). Finally, cancer cells cross the endothelium to complete TEM. In our assays, HUVECs are not stimulated with any inflammatory cytokines and therefore expressed very low or undetectable levels of the leucocyte adhesion molecules ICAM‐1 or VCAM‐1. Stimulation of HUVECs with TNF‐α did not increase adhesion of PC3 cells (data not shown), whereas this is well known to be required for leucocyte attachment to ECs (Daniel and van Buul, 2013).

Supplementary data related to this article can be found online at http://dx.doi.org/10.1016/j.molonc.2015.01.004.

**Figure 1:**
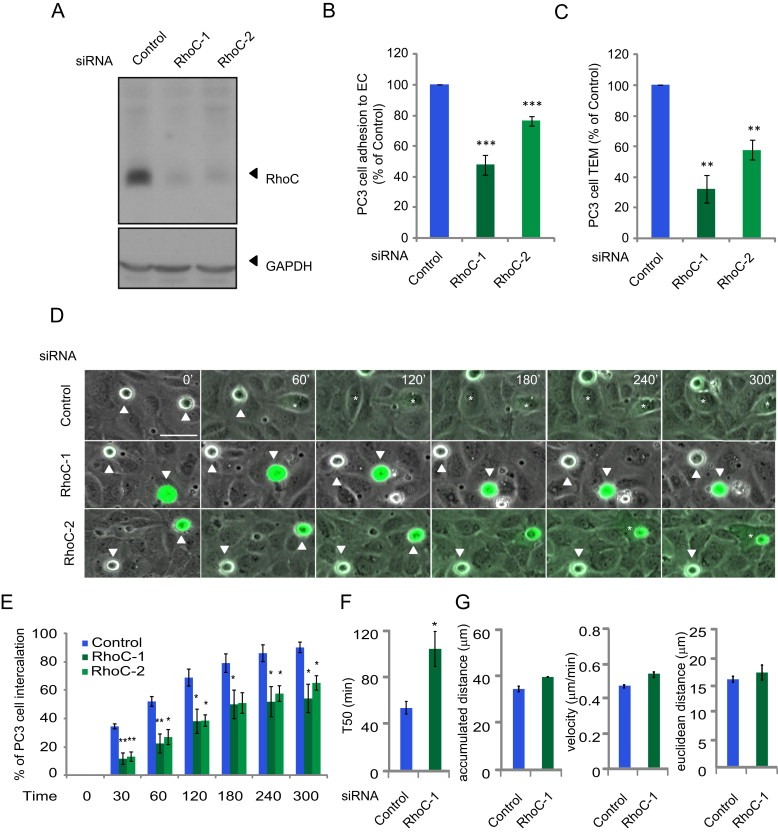
RhoC regulates cancer cell adhesion to and intercalation between endothelial cells.(A) Lysates of PC3 cells transfected with indicated single siRNAs were immunoblotted with antibodies to RhoC, and GADPH as a loading control. (B) CFSE‐labelled PC3 cells transfected with the indicated siRNAs were added to HUVECs for 15 min and % adhesion relative to control siRNA‐transfected cells determined. Values are means ± SEM (n = 3); ***p < 0.001. (C) CFSE‐labelled PC3 cells transfected with the indicated siRNAs were added to HUVECs grown on a transwell insert for 8 h and % transmigrated cells relative to control siRNA‐transfected cells determined. Data are expressed as % of total cells± SEM (n = 3); **p < 0.01. (D) CFSE‐labelled‐PC3 cells transfected with the indicated siRNAs were filmed for 300 min on HUVECs. Asterisks mark PC3 cells that intercalate; white arrows indicate cells that have not intercalated. Scale bar, 50 μm. (E) Graphs show time of intercalation for individual cells transfected with the indicated siRNAs. Cells were filmed on HUVECs for 300 min. In each experiment ≥100 cells were analysed in at least 3 fields. Data are expressed as % of total cells. Values are means ± SEM (n = 3); **p < 0.01, *p < 0.05. See also Movie S1. (F) Time when 50% of PC3 cells have intercalated within ECs (T50). Values are means ± SEM (error bars; n ≥ 3); *p < 0.05. (G) Total distance migrated (left), velocity of cells (middle) and Euclidean distance migrated (right) of PC3 cells on top of ECs before intercalation.

To test the role of RhoC in these sequential steps of cancer cell interaction with ECs, PC3 cells were transfected with 2 different siRNAs targeting RhoC (Figure 1A). We have previously shown that these RhoC siRNAs do not alter the expression of RhoA or Rac1 (Vega et al., 2011). First, RhoC depletion with either of these 2 siRNAs reduced PC3 cell adhesion to ECs (Figure 1B). Second, RhoC depletion strongly inhibited PC3 cell TEM in Transwell assays (Figure 1C). Third, as monitored by time‐lapse microscopy, RhoC‐depleted cells remained round on top of ECs for a longer period of time compared to control cells, and were significantly delayed in their intercalation between ECs (Figure 1D, E and Movie S1). The intercalation T_50_ is defined as the time when 50% of a given cancer cell population has intercalated (Reymond et al., 2012b). RhoC‐depleted PC3 cells had a higher T_50_ (105 min) compared to control cells (50 min) (Figure 1F).

Although intercalation was delayed, this was not due to impaired migration of PC3 cells on ECs: their velocity as well as the distance migrated on ECs did not differ between RhoC‐depleted cells and control cells, (Figure 1G). By contrast, we found that RhoA depletion not only inhibits PC3 cell adhesion to ECs but also reduces their velocity on top of ECs (Reymond et al., 2012a). This indicates that RhoA and RhoC act in different ways to affect cancer cell: EC interaction even though they both contribute to the initial step of cancer cell adhesion to ECs.

### 3.2 RhoC depletion affects cancer cell opening of EC junctions

**Figure 2:**
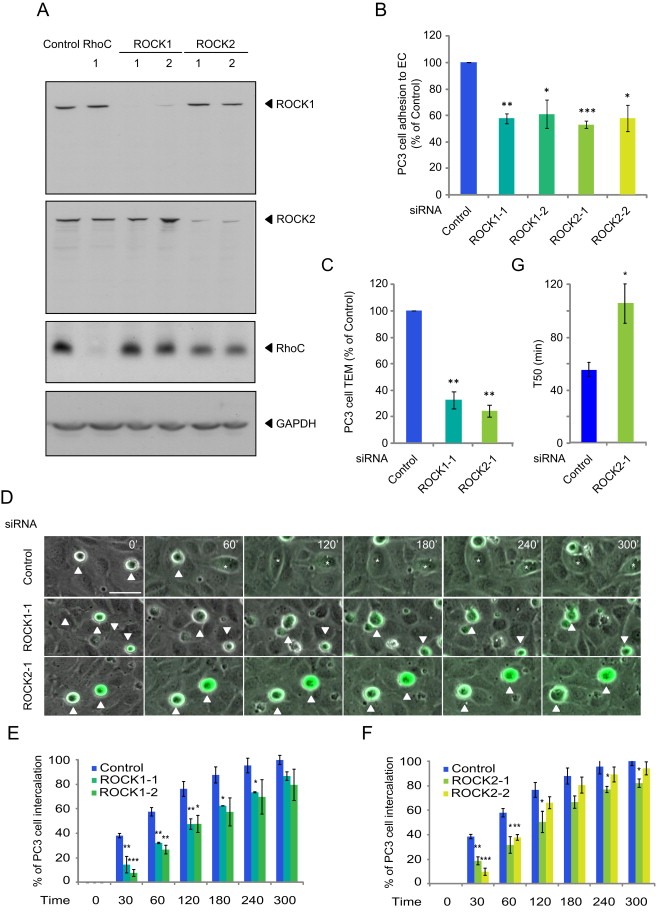
ROCK1 and ROCK2 regulate cancer cell interaction with endothelial cells. (A) Lysates of PC3 cells transfected with the indicated siRNAs were immunoblotted as shown. (B) CFSE‐labelled PC3 cells transfected with the indicated siRNAs were added to HUVECs for 15 min and % adhesion relative to control determined. Values are means ± SEM (n = 3); ***p < 0.001, **p < 0.01, *p < 0.05. (C) CFSE‐labelled PC3 cells transfected with the indicated siRNAs were added to HUVECs grown on a transwell insert for 8 h and % transmigration relative to control determined. Data are expressed as % of control. (D) CFSE‐labelled‐PC3 cells transfected with the indicated siRNAs were filmed for 300 min on HUVECs. Asterisks mark PC3 cells that intercalate; white arrow indicates a cell that does not intercalate. Scale bar, 50 μm. (E–F) Graphs show time of intercalation for individual cells transfected with the indicated siRNAs. Cells were filmed on HUVECs for 300 min. In each experiment, ≥100 cells were analysed in at least 3 fields. Data are expressed as % of total cells. (G) Time when 50% of PC3 cells have intercalated between ECs. Values are means ± SEM (n = 3); **p < 0.01, *p < 0.05. See also Movie S2.

**Figure 3:**
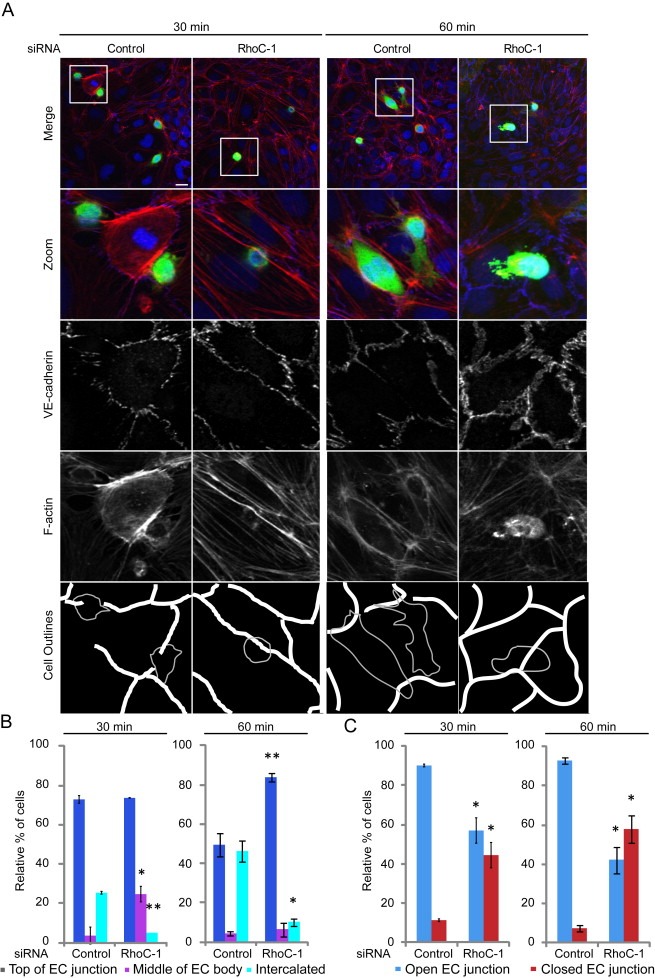
RhoC depletion delays endothelial cell junction opening.(A) CFSE‐labelled PC3 cells were added to HUVECs for 30 or 60 min then stained for VE‐cadherin and F‐actin. Cell outlines are shown. Gaps in the endothelial monolayer by the control PC3 cells are indicated in the ‘Cell Outlines’. Scale bar, 20 μm. (B) Quantification of sites of cancer cell adhesion with respect to EC junctions. (C) Status of EC junctions near cancer cell adhesion sites. Data are expressed as % of total cells analysed; ≥50 cells/experiment. Values are means ± SEM (n = 3); **p < 0.01, *p < 0.05.

We then investigated how RhoC affects the interaction of cancer cells with ECs. By 30 min after addition to ECs, most control PC3 cells were localized on top of EC junctions, as we previously described (Figure 2A; (Reymond et al., 2012a)). Fewer RhoC‐depleted cells compared to control cells were detected on ECs, reflecting their reduced adhesion (data not shown). In addition, RhoC‐depleted cells were less frequently localized on top of EC junctions and a significant number of them were localized in the middle of an EC body with no contact with EC junctions (Figure 2A, B). Control cells that were localized on top of EC junctions frequently induced the opening of these junctions, as determined by the local loss of VE‐cadherin staining and the creation of small gaps between ECs (Reymond et al., 2012a). By contrast, RhoC‐depleted cells rarely induced EC junctional opening at either 30 or 60 min after addition to ECs (Figure 3A, C). This reduced ability to induce junctional disassembly explains why RhoC‐depleted cells show impaired intercalation and subsequent TEM.

### 3.3 ROCKs affect PC3 cell adhesion to ECs and transmigration similarly to RhoC

**Figure 4:**
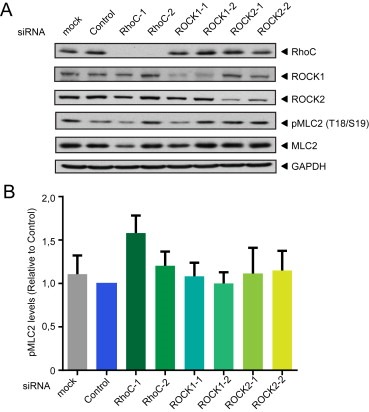
RhoC, ROCK1 and ROCK2 do not affect myosin light chain phosphorylation in PC3 cells. PC3 cells treated with transfection reagent alone (Mock) or transfected with control siRNA (Control) or the indicated siRNAs targeting RhoC, ROCK1 or ROCK2 were lysed, and lysates were immunoblotted with the indicated antibodies. (A) Representative immunoblot. (B) Results of three independent experiments. There was no significant difference between any conditions (One‐way ANOVA and Tukey post‐test).

**Figure 5:**
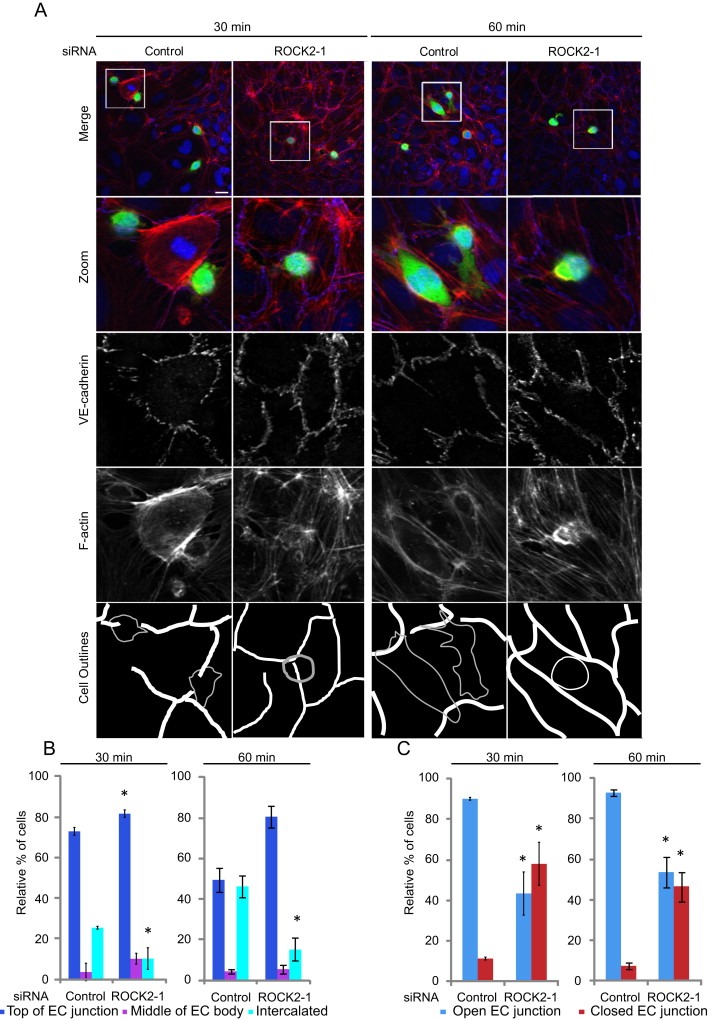
ROCK1 and ROCK2 depletion delays EC junction opening.(A) CFSE‐labelled PC3 cells were added to HUVECs for 30 or 60 min then stained for VE‐cadherin and F‐actin. Cell outlines are shown. Gaps in the endothelial monolayer by the control PC3 cells are shown in ‘Cell Outlines’. Scale bar, 20 μm. (B) Quantification of sites of cancer cell adhesion with respect to EC junctions. (C) Status of EC junctions near cancer cell adhesion sites. Data are expressed as % of total cells analysed; ≥ 50 cells/experiment. Values are means ± SEM (n = 3); *p < 0.05.

ROCKs are the best characterized targets for the three closely related Rho proteins, RhoA, RhoB and RhoC (Julian and Olson, 2014; Wheeler and Ridley, 2004). The two ROCKs, ROCK1 and ROCK2, were depleted with 2 different siRNAs for each kinase (Figure 3A). As observed for RhoC (Figure 1), ROCK1 and ROCK2 depletion inhibited PC3 cell adhesion to ECs (Figure 3B) and strongly reduced TEM (Figure 3C). ROCK1 and ROCK2 depletion also induced a delay of cancer cell intercalation within EC monolayers (Figure 3D–G and Movie S2). Similar to RhoC‐depleted cells, the migration speed of ROCK1‐ or ROCK2‐depleted cells on top of ECs was not altered and the T_50_ of intercalation was increased (Figure 3G, data not shown). Consistent with ROCKs acting on the same pathway as RhoC, fewer ROCK1‐ (data not shown) and ROCK2‐depleted cells localized above EC junctions, and they had a reduced ability to induce junction opening compared to control cells at 30 and 60 min (Figure 4A–C). Our data suggest that ROCK1 and ROCK2 might act downstream of RhoC to regulate cancer cell adhesion to and transmigration across ECs. ROCKs are well known to stimulate phosphorylation of myosin light chain (MLC), and thereby stimulate contractility (Julian and Olson, 2014). However, depletion of RhoC, ROCK1 or ROCK2 did not reduce levels of phosphorylated MLC in PC3 cells (Figure 5), and thus it is unlikely that the RhoC/ROCK pathway regulates TEM via effects on actomyosin contractility.

Supplementary data related to this article can be found online at http://dx.doi.org/10.1016/j.molonc.2015.01.004.

### 3.4 RhoC is required for cancer cell spreading on vascular ECs *in vivo*

**Figure 6:**
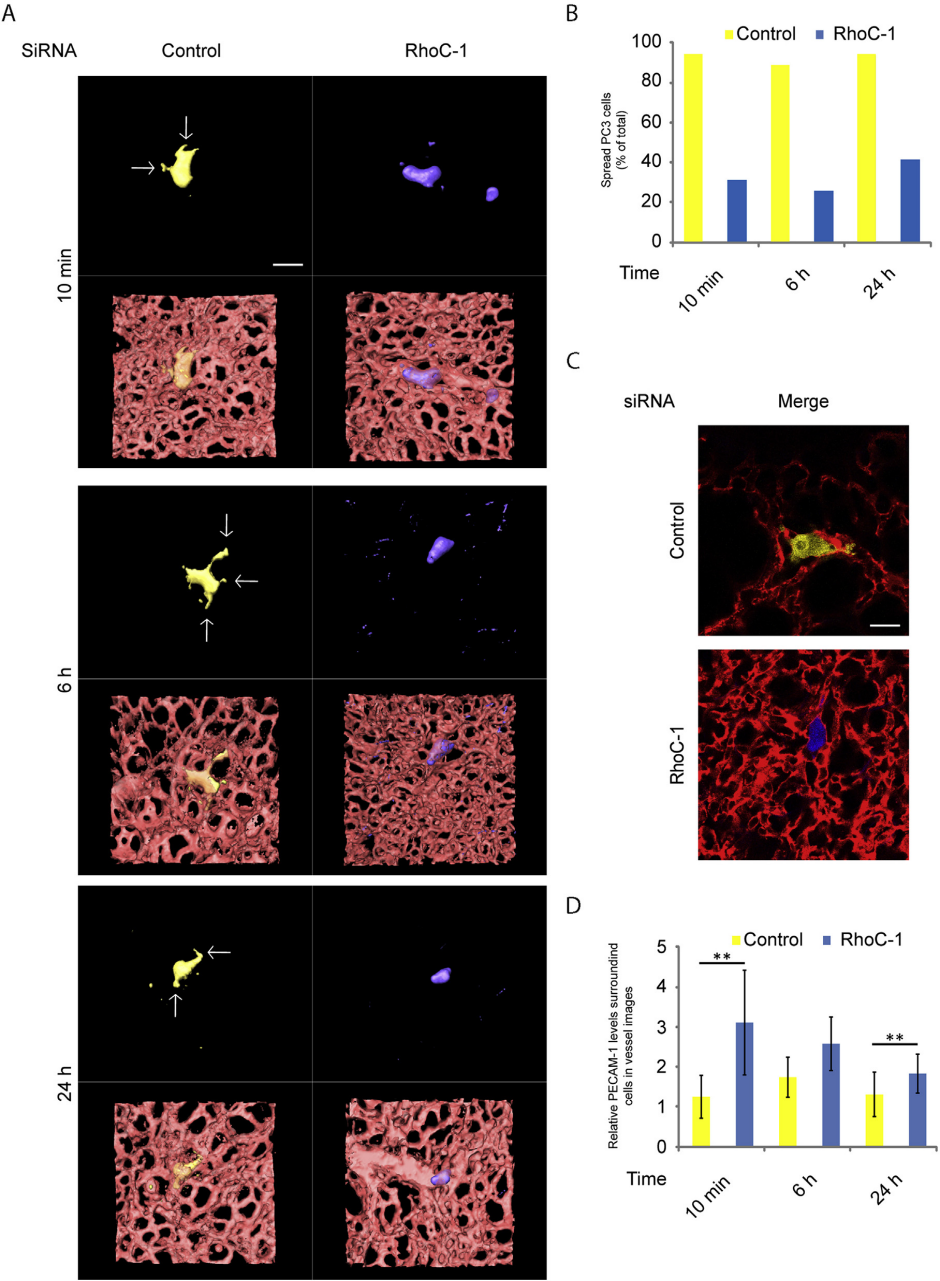
RhoC regulates PC3 cancer cell spreading on lung vascular endothelial cells in vivo.CFP‐PC3 cells transfected with RhoC siRNA and YFP‐PC3 cells transfected with control siRNA were co‐injected into mice. (A) 3D reconstructions of representative confocal 3D stacks of cells in the lung vasculature. Arrows indicate cancer cell protrusions along the vessels. Scale bar, 20 μm. (B) Quantification of cells with protrusions in the lung vasculature. At least 50 single cells per condition were analysed from at least 3 independent experiments. Data are expressed as % of total number of cells analysed. (C–D) CFP‐PC3 cells transfected with siRNA‐1 targeting RhoC and YFP‐PC3 cells transfected with control siRNA (Control) were injected simultaneously in mice. Representative confocal images acquired 6 h after PC3 cell injection and used for the analysis of endothelial PECAM‐1 staining around cancer cells. ECs were stained by tail‐vein injection of PE‐labelled anti‐PECAM‐1 antibody 10 min before acquisition of images (C). Quantification of pixel intensities of PECAM‐1 staining around PC3 cells at the indicated time points (D) ; n = 10 cells per condition, **p < 0.01.

To investigate whether RhoC contributes to interaction of cancer cells with ECs *in vivo*, PC3 cells were analysed in blood vessels in the lung. Control siRNA‐transfected YFP‐PC3 cells and RhoC‐depleted CFP‐PC3 cells were introduced simultaneously into the vena cava or the tail vein of mice. By 10 min after injection, most control YFP‐PC3 cells detected in the lungs extended single or multiple protrusions on ECs (Figure 6A), as we described previously (Reymond et al., 2012a). This phenotype was also observed at 6 and 24 h after injection (Figure 6A, B). In contrast, most RhoC‐depleted CFP cells remained rounded or assumed the tubular shape of the surrounding blood vessels. They rarely extended protrusions at any time‐point after injection: 10 min, 6 h or 24 h (Figure 6A, B, Movies S3 and S4). To evaluate the interaction of cancer cells with ECs in more detail, we analysed the localization of endothelial PECAM‐1 around cancer cells in the lung blood vessels as previously described (Reymond et al., 2012a). The pixel intensity of PECAM‐1 staining surrounding cancer cells in blood vessels was higher around RhoC‐depleted cells compared to control cells at 10 min after cell injection (Figure 6C, D). This suggests that control cells were already strongly attached to ECs and thus prevented access of PECAM‐1 antibodies. This difference in PECAM‐1 staining was also observed at 6 or 24 h (Figure 6D), implying that RhoC‐depleted cells remain only loosely attached to ECs compared to control cells *in vivo*. Since RhoC‐depleted cells remained mostly rounded within the vessels, our results are consistent with a role for RhoC in EC interaction *in vivo*.

Supplementary data related to this article can be found online at http://dx.doi.org/10.1016/j.molonc.2015.01.004.

### 3.5 RhoC depletion reduces early lung retention of cancer cells and metastasis *in vivo*

**Figure 7:**
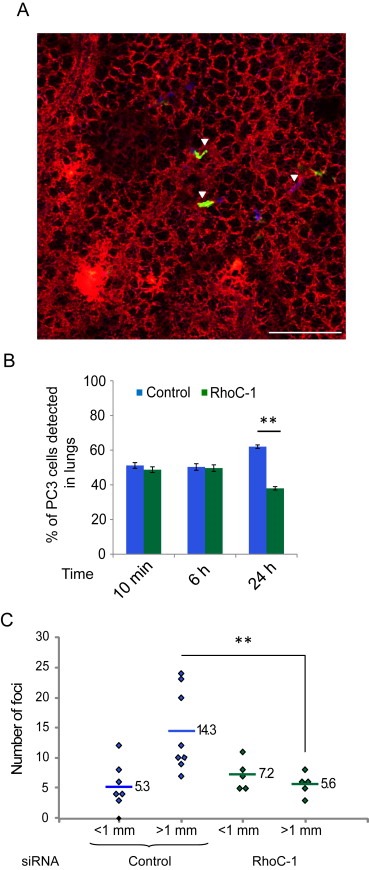
RhoC regulates PC3 cancer cell retention in the lungs and experimental metastasis formation in vivo. (A) Representative 2D image used to monitor the ratio between siRNA control‐transfected YFP‐PC3 and RhoC‐depleted CFP‐PC3 cells. Scale bar, 200 μm. (B) Number of cells in the lungs. 60 random independent fields were analysed and cell number was scored from 3 independent experiments. Data are expressed as % of total cells analysed. Values are means ± SEM; **p < 0.01. (C) Effects of RhoC depletion on experimental metastasis formation. Mice were injected intravenously with PC3 cells transfected with control siRNA or RhoC‐1 siRNA. Quantification of metastatic foci on the lung surface (8 mice for control, 5 mice for RhoC siRNA). Each dot represents foci from one mouse; lines and numbers indicate means; **p < 0.01.

Most of experimentally injected cancer cells die by apoptosis during the first 24–48 h in the lung vasculature (Mehlen and Puisieux, 2006). We therefore investigated whether transient RhoC depletion affected PC3 cell retention in the lung vasculature by assessing the ratio between control YFP‐cells and RhoC‐depleted CFP‐cells detected in the lung blood vessels at different time points (Figure 7A, B). At 10 min and 6 h after injection, the two populations were equally present in the lungs (50/50 ratio). However, by 24 h after injection, more control cells (60%) than RhoC‐depleted cells (40%) were detected. This suggests that the defect in spreading on ECs due to RhoC depletion might reduce cancer cell retention in the lungs.

RhoC was initially described to contribute to melanoma metastasis in mouse models (Clark et al., 2000), and has since been described to participate in cancer spreading in other types of cancer such as prostate (Iiizumi et al., 2008) and breast cancer (Rosenthal et al., 2011). The transient depletion of the Rho family GTPase Cdc42 inhibited the formation of experimental metastases in the lung (Reymond et al., 2012a). The effect of transient RhoC depletion on the metastatic potential of PC3 cells was therefore assessed. Mice injected with PC3 cells transiently depleted of RhoC with siRNA developed less tumour foci in their lungs compared to mice injected with control siRNA‐transfected cells (Figure 7C). Altogether, our *in vitro* and *in vivo* data show that RhoC is important for cancer cell interaction with ECs, and imply that this step is critical for cancer cell retention in blood vessels and subsequent metastasis formation. Previous studies showed that RhoC does not have any effect on cell proliferation *in vitro* or on tumour growth in mice but affects metastasis (Iiizumi et al., 2008). Our results suggest that RhoC predominantly affects lung metastasis by affecting the step of cancer cell interaction with ECs.

## 4 Discussion

RhoC was one of the first genes reported to stimulate cancer cell metastasis in mice (Clark et al., 2000), and subsequent studies have linked its expression to metastasis in a wide range of human cancers (Vega and Ridley, 2008). We report here that RhoC is important for cancer cell interaction with ECs during TEM, which could explain its central role in metastasis. Our results show that RhoC regulates the extension of protrusions by cancer cells along ECs *in vivo*, which correlates with effects on early cancer cell retention in the lungs and long‐term experimental metastasis. This supports a model where the early attachment of cancer cells to ECs is a critical step for the subsequent growth of metastases.

We previously showed that the reduced adhesion of Cdc42‐depleted cancer cells to ECs was due to decreased β1 integrin expression (Reymond et al., 2012a). However, unlike Cdc42, RhoC did not affect β1 integrin levels (data not shown). RhoC depletion was previously reported to lower α5 integrin expression in melanoma cells (Arpaia et al., 2012). α5 integrin interacts specifically with β1 integrin, and α5β1 attaches to fibronectin (Humphries, 2000). We have found that RhoC depletion in PC3 cells increased cell spreading on fibronectin (Vega et al., 2011), and does not affect their adhesion to fibronectin (unpublished data) making it unlikely that RhoC affects α5β1 levels in these cells.

The reduced adhesion of RhoC‐depleted cells to ECs could affect their ability to induce EC junction opening. It is also possible that RhoC regulates the expression of cell surface receptors involved in the opening of EC junctions. We have previously shown that RhoC depletion inhibits PC3 cell migration and invasion through Matrigel (Vega et al., 2011). Moreover, in prostate cancer cells, RhoC activates matrix metallo‐proteinases 2 and 9 (MMP2 and MMP9) *in vitro* (Iiizumi et al., 2008), which could contribute to invasion. This implies that RhoC would affect cancer cell invasion through the basement membrane after the TEM step as well as cancer cell: EC interactions. This would explain its strong effect on metastasis (Iiizumi et al., 2008; Rosenthal et al., 2011; Vega and Ridley, 2008).

Interestingly, cancer cell protrusions seem to give an advantage for cell survival once cells have extravasated and need to grow in the vessel vicinity (Shibue et al., 2012). It will be interesting to observe if RhoC or Cdc42 that we describe to be important for cancer cell: EC interaction give an advantage for their survival in the microenvironment surrounding the vessels. In a zebrafish model, RhoC works in cooperation with VEGF to enable cancer cell intravasation: RhoC increases the ability of cells to form specialized invadopodia to protrude through vascular EC openings (Stoletov and Klemke, 2008; Stoletov et al., 2007). We and others therefore put RhoC at the centre of a network that will regulate protrusion and thus promote metastasis.

NK cells are present in the SCID mice used in our studies, and contribute to anti‐tumoral protection in the lungs (Yang et al., 2006). It is possible that the depletion of RhoC sensitizes cancer cells to apoptotic signals induced by NK cells, which could explain the reduced levels of RhoC‐depleted cells compared to control cells in the lung at 24 h after injection. Interestingly, platelets contribute to cancer cell spreading on ECs during early metastatic colony formation and they protect cancer cells from NK cells in the lung by attaching to them very soon after injection (Im et al., 2004). However, platelets detach from cancer cells approximately 6 h after their interaction, suggesting that RhoC does not act by regulating interactions of cancer cells with platelets.

We identify here the RhoC/ROCK pathway as a strong candidate for therapeutic targeting to reduce cancer metastasis, and indeed ROCK inhibitors have previously been shown to reduce experimental metastasis (Itoh et al., 1999). Even though ROCKs are best known for their effects on MLC phosphorylation and actomyosin contractility, we have found that this pathway is not altered by RhoC/ROCK depletion in PC3 cells. ROCKs also signal downstream of RhoA. We previously reported that RhoA depletion reduces cancer cell adhesion to ECs and delays cancer cell intercalation (Reymond et al., 2012a); however, RhoA depletion reduced cancer cell velocity on top of ECs before intercalation, which was not the case when RhoC or ROCKs were depleted. The effects of RhoC/ROCK depletion on cancer cell:EC interaction are thus different from RhoA depletion. This is consistent with our previous observations that RhoA and RhoC induce distinct phenotypes in cancer cells (Vega et al., 2011).

Cancer cell adhesion is central to the metastasis process since it is linked to survival, growth, interaction with immune cells and vascular ECs (Reymond et al., 2013). Metastatic tumour cell attachment to ECs has been shown to induce the endothelial activation markers VCAM‐1 and VAP‐1 (Ferjancic et al., 2013). It will be interesting to assess the role of RhoC in this context. Anti‐adhesion therapies show promising potential and could be used to target specifically cancer cells during tumour dormancy or relapse, as well as before cells actually reach secondary targets early during the metastasis process.

## Author contributions

N.R. and A.J.R. conceived the project, N.R. designed and performed experiments and analysed raw data. J.H.I designed and carried out mouse experiments. R.G., P.R., M.S. and A.C. assisted with experiments, S.C. analysed PECAM‐1 localisation. R.J.M designed mouse experiments. N.R and A.J.R wrote the manuscript.

## Supporting information

The following are the Supplementary material related to this article:

Movie S1 RhoC depletion delays cancer cell intercalation. Relates to Figure 1C. CFSE‐labelled‐PC3 cells transfected with control siRNA or siRNAs RhoC‐1 or RhoC‐2 were filmed on HUVECs by timelapse microscopy. Frames were taken every 5 min for 300 min

Movie S2 ROCK1 and ROCK2 depletion delays cancer cell intercalation. Relates to Figure 3D. CFSE‐labelled‐PC3 cells transfected with the control siRNA or siRNAs ROCK1‐1 or ROCK2‐1 were filmed on HUVECs by timelapse microscopy. Frames were taken every 5 min for 300 min

Movie S3 Morphology of PC3 cells within lung blood vessels. Relates to Figure 6A. PC3 cells expressing YFP (yellow) were transfected with a control siRNA. Cells were injected in the tail vein in SCID mice. After 6 h, a PECAM‐1 antibody was injected to stain blood vessels, then the lungs were isolated and analysed by confocal microscopy. 3D images were processed using Amira software.

Movie S4 Morphology of RhoC‐depleted PC3 cells within lung blood vessels.Relates to Figure 6A. PC3 cells expressing CFP (blue) were transfected with siRNA RhoC‐1. Cells were injected in the tail vein in SCID mice. After 6 h, a PECAM‐1 antibody was injected to stain blood vessels (red), then the lungs were isolated and analysed by confocal microscopy. 3D images were processed using Amira software.
